# Bridge to surgery using a self-expandable metallic stent for stages II–III obstructive colorectal cancer

**DOI:** 10.1186/s12893-020-00847-z

**Published:** 2020-08-20

**Authors:** Katsuya Ohta, Masakazu Ikenaga, Masami Ueda, Kiyotsugu Iede, Yujiro Tsuda, Shinsuke Nakashima, Takashi Nojiri, Jin Matsuyama, Shunji Endo, Jun Murata, Ichizo Kobayashi, Masahiko Tsujii, Terumasa Yamada

**Affiliations:** 1 Gastroenterological Surgery, Higashiosaka City Medical Center, Osaka, Japan; 2 Department of Gastroenterological Surgery, Kindai University Nara Hospital, 1248-1 Otoda-cho, Ikoma-city, Nara, 630-0293 Japan; 3 Thoracic Surgery, Higashiosaka City Medical Center, Osaka, Japan; 4 Digestive Surgery, Kawasaki Medical School, Okayama, Japan; 5 Gastroenterology, Higashiosaka City Medical Center, Osaka, Japan

**Keywords:** Bowel obstruction, Colorectal cancer, Self-expandable metallic stent

## Abstract

**Background:**

Bridge to surgery (BTS) using a self-expandable metallic stent (SEMS) for the treatment of obstructive colorectal cancer improves the patient’s quality of life. This study aimed to examine prognostic factors of obstructive colorectal cancer.

**Methods:**

We analyzed stage II-III resectable colon cancer cases (Cur A) retrospectively registered between January 2005 and December 2017. Overall, 117 patients with Cur A obstructive colorectal cancer were evaluated: 67 of them underwent emergency surgery (ES Group) and 50 of them after BTS with SEMS placement (BTS group). We compared surgical results and prognoses between the two groups.

**Results:**

A total of 50 patients underwent endoscopic SEMS placement, which technical success of 96% and morbidity rate of 18%. Primary anastomosis rates were 77.6% in ES and 95.7% in BTS (*p* <  0.001); postoperative complication, 46.3% in ES and 10.5% in BTS (*p* <  0.001); pathological findings of lymphatic invasion, 66.7% in ES and 100% in BTS (*p* <  0.001); venous invasion were 66.8% in ES and 92% in BTS (*p* = 0.04); and recurrence of 25.4% in ES and 39.1% in BTS. The 3-year overall survival was significantly different between two groups (ES, 86.8%:BTS, 58.8%), BTS is worse than ES (log-rank test; *p* <  0.001). Venous invasion independently predicted worsened recurrence-free and overall survival.

**Conclusions:**

The vascular invasiveness was correlated with tumor progression after SEMS placement, and the survival rate was lower in BTS. SEMS potentially worsens prognostic outcomes in stage II–III obstructive colorectal cancer.

## Background

Colorectal cancer (CRC) remains the leading cause of cancer-related deaths worldwide because several patients are initially diagnosed during advanced stages [1]. Approximately 8–13% of patients with CRC were diagnosed with acute colonic obstruction [2–4]. Severe malignancy with bowel obstruction needs urgent surgical intervention, which includes primary lesion resection and stoma creation, leading to increased morbidity and mortality and a potential failure to achieve complete oncological resection [5, 6].

An endoscopic procedure with self-expandable metallic stent (SEMS) is an acceptable bridge to surgery (BTS) treatment for acute colonic obstruction [7–9]. Preoperative SEMS placement provides an opportunity to perform medical resuscitation, comorbidity optimization, bowel preparation, tumor staging, and observation of proximal lesions [10]. The procedure prevents high-risk emergency surgeries and increase oncological resection and primary anastomosis rates [10, 11]. After the inclusion of colonic SEMS placement as BTS in the coverage of the National Health Insurance in Japan, several physicians joined the Colonic Stent Safety Procedure Research Group and developed skills to provide safe treatment. The largest multicenter prospective study demonstrated the feasibility and safety of SEMS placement as BTS in patients with malignant colorectal obstruction [12].

The oncological safety and minimal invasiveness of this procedure have confirmed that SEMS placement as a bridge to elective surgery is not recommended as a standard treatment for symptomatic left-sided malignant colonic obstruction [13, 14]. Several studies reported that prognostic factors of malignant colonic obstruction in SEMS placement had oncological disadvantages compared with those in emergency surgery (ES) [15, 16]. In contrast, several trials showed that SEMS placement as a bridge to elective surgery did not improve the survival rates [17–20]. How SEMS placement worsens prognostic outcomes remains unclear [21, 22].

This study aimed to evaluate the induction of curative surgery in patients with malignant colorectal obstruction after a SEMS placement and its long-term results and prognostic factors postoperatively compared to patients without SEMS placement. We demonstrated prognostic factors and overall survival (OS) and recurrence-free survival (RFS) rates for curative surgery after a SEMS placement.

## Methods

### Patients

Medical records of patients who underwent primary colorectal resection at Higashiosaka City Medical Center between January 2005 and December 2017 were reviewed. All participants provided written informed consent. Oral intake and symptoms before and after SEMS placement were assessed in Table 1 using the ColoRectal Obstruction Scoring System (CROSS). From 2012 to 2015, we recruited patients with all class of CROSS as stent insertion candidate. From 2015, we excluded patients with CROSS 3 and 4 based on up-dated stent insertion guideline [10]. Malignant colorectal obstruction was diagnosed through clinical examination, CROSS, radiography, and computed tomography. Surgery was performed using three approaches: ES comprised laparotomy, lymph node dissection as possible, and primary anastomosis on the same day between 2005 and 2011. BTS after SEMS placement comprised standby laparoscopy, D3 lymph node dissection, and primary anastomosis since January 2012. Overall, 117 patients with Stage II-III (Cur A) obstructive colorectal cancer were evaluated: 67 of them underwent emergency surgery as ES Group and 50 of them after BTS with SEMS placement as BTS group. We compared surgical results and prognoses between the two groups.

**Table 1:** The ColoRectal Obstruction Scoring System (CROSS)

Patient’s symptom and their condition of an oral intake	CROSS
Solid meal, low residue, and full diet without symptom	4
Solid meal, low residue, and full diet with symptom	3
Liquid or enteral nutrient intake	2
No oral intake	1
Requiring continuous decompression	0

### SEMS devices and the procedure

Patients were endoscopically treated with placement of an uncovered WallFlex enteral colonic stent (Boston Scientific Corporation, Natick, MA, USA) or Niti-S enteral colonic uncovered stent (Taewoong, Inc., Gimpo, South Korea). Placements were performed as presented in the pre-introduction publicity announcement. Placement details were mentioned on the website as a brief guideline [10]. Obstruction structures were determined using a guide wire, and a contrast tube was inserted into the proximal colorectal lumen. Obstructions were measured using contrast agents, and then the endoscopist determined the number, size, and type of stent. Pathological biopsies were recommended after SEMS. Locations and intraluminal or extraluminal marking using an endoscopic clip were recommended via visual recognition of the endoscopist. Dilatation of the colonic obstruction before SEMS placement was generally not allowed.

### Histological findings

Paraffin-embedded specimens were obtained from a cohort of 117 patients diagnosed by the Union for International Cancer Control stage II–III.

### Survival definitions

OS was defined as the duration from surgery to any death or last follow-up. Diagnosis of recurrence was calculated based on RECIST 1.1 according to the chemotherapy criteria [23]. RFS was defined as the duration from surgery to any recurrence includes local recurrence or distant metastasis.

### Statistical analysis

Student’s *t*-test and Wilcoxon test for continuous variables and the χ^2^ and Fisher’s exact tests for categorical variables were conducted. Survival curves were generated using the Kaplan–Meier method and compared using a log-rank test. Univariate and multivariate survival analyses were performed using the Cox proportional hazards regression model. All statistical analyses used JMP (version 8.01, SAS Institute, Cary, NC) or statistical scripting language R (http;//www.r-project.org/). *P*-values of ≤0.05 (two-sided) were considered statistically significant. This prognostic study complied with the reporting recommendations for Tumor Marker Prognostic Studies [24].

## Results

A total of 50 patients underwent endoscopic SEMS placement, which was technically safe for malignant colorectal obstruction, with the technical success rate of 96%. The clinical success rate was 92%, and the patient’s symptoms and oral intake dramatically improved after the SEMS placement, shown in Table 2. A total of 117 patients were reviewed: 67 and 50 patients underwent ES and BTS, respectively, as shown in Table 3. Baseline clinical characteristics were balanced between the two groups. Moreover, 79.1% (53 cases) of patients underwent ES on the same day as in open surgery. The median waiting period for surgery was 14 days for BTS. The primary anastomosis ratios were 77.6% in ES and 95.7% in BTS (*p* <  0.001). Postoperative complication rates were 46.3% in ES and 10.5% in BTS (*p* <  0.001). Postoperative hospital stay was shorter in BTS (11 days) compared to ES (17 days) (*p* = 0.002). Patients with obstructive CRC showed significant improvement in postoperative complication rate and hospital stay with SEMS placement. Operative procedures were dramatically changed, and the primary anastomosis rate improved after the SEMS placement.

**Table 2:** Baseline characteristics and outcomes of endoscopic SEMS placement

		BTS (*n* = 50)
Gender	Male/Female	22 / 28
Age	Median (range)	73 (44–90)
Location	Cecum	0
	Ascending	8
	Transverse	8
	Descending	6
	Sigmoid	20
	Rectum	8
Length of obstruction	Median (range; cm)	3 (2–8)
Technical success		48 (96%)
Procedure	Through the scope	42
	Through the wire	6
Stenting	Wall Flex 6/ 9 cm	33/ 3
	Niti-S 6/ 8/ 10 cm	7/ 4/ 1
Morbidity	Overall	5 (18%)
	> C-D^a^ III	1 (2%)
Mortality	C-D^a^ V	1 (2%)
Clinical success		46 (92%)
	CROSS	Before / After
	4	1/ 46
	3	13/ 0
	2	4/ 2
	1	14/ 1
	0	18/ 1

^a^ Clavien–Dindo classification

**Table 3:** Comparison of baseline characteristics in patients undergoing emergency surgery and bridge to surgery

		ES (*n* = 67)	BTS (*n* = 50)	*p*
Gender	Male/Female	35 / 32	22 / 28	0.377
Age	Median (range)	69 (33–93)	73 (44–90)	0.106
Location	Cecum	0	0	0.657
	Ascending	6	8	
	Transverse	10	8	
	Descending	14	6	
	Sigmoid	25	20	
	Rectum	12	8	
Type of operation	Standby	14 (21%)	44 (88%)	< 0.001
	Emergency	53 (79%)	6 (12%)	
Duration to operation	Median (range; days)	0 (0–27)	14 (0–67)	< 0.001
Surgical Procedure	Laparotomy	67 (100%)	21 (39%)	–
	Laparoscopy	0	29 (61%)	
Time	Median (range; min)	203	215	0.808
		(123–508)	(99–648)	
Blood loss	Median (range; mL)	324 (0–526)	69 (0–2495)	< 0.001
Stoma creation		15 (22%)	3 (14%)	< 0.001
Morbidity	30-day complication	31 (46%)	5 (10%)	< 0.001
	> C-D^a^ III	6 (9%)	2 (4%)	< 0.001
	Anastomostic leakage	4 (22%)	1 (2%)	< 0.001
Hospital Stay	Median (range; days)	17 (6–120)	11 (7–140)	0.002

^a^Clavien–Dindo classification

**Fig. 1:**
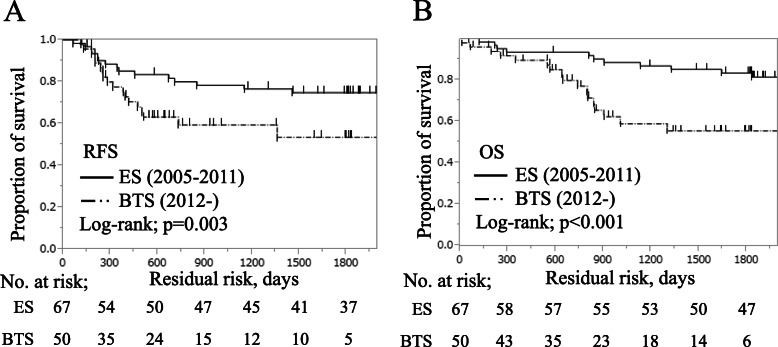
Kaplan–Meier survival curves in patients undergoing emergency surgery vs. bridge to surgery. **a** Recurrence-free survival. **b** Overall survival

**Fig. 2:**
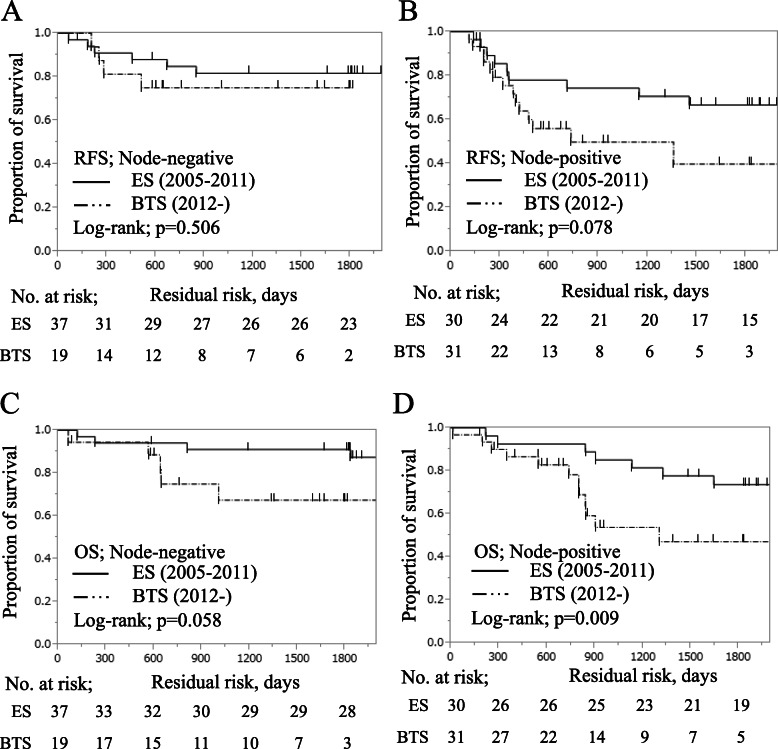
Kaplan–Meier survival curves in patients undergoing emergency surgery vs. bridge to surgery. **a** RFS; node-negative patients. **b** RFS; node-positive patients. **c** OS; node-negative patients. **d** OS; node-positive patients

The pathological tissue type accounted for 96.4% of differentiated types shown in Table 4. Tumor depth was similar between the two groups. Lymphatic vessel invasion ratios were 66.7% in ES and 100% in BTS (*p* <  0.001), and venous invasion ratios were 66.8% in ES and 92% in BTS (*p* = 0.038). Recurrence rates were 25.4% (17 cases) in ES and 39.1% (18 cases) in BTS. Node-negative patients (stage II) more frequently had lung metastasis (54.5%), whereas node-positive patients (stage III) more frequently had liver metastasis (41.7%). In the Kaplan–Meier survival analysis in Fig. 1a, the 3-year RFS was significantly different between the two groups (ES, 76.6%; BTS, 59.4%), which was significantly low in BTS than that in ES (log-rank test; *p* = 0.003). The 3-year OS rate was also significantly different between the two groups: ES, 86.8%; BTS, 58.8%; *p* <  0.001, shown in Fig. 1b. The relationship between lymph node metastasis and SEMS placement was also evaluated. The pathological node-negative (stage II) 3-year RFS rate was not different between the two groups (ES, 81.6%; BTS, 75.0%) as shown in Fig. 2a. In contrast, the pathological node-positive (stage III) 3-year RFS rate was different between the two groups (ES, 70.7%; BTS, 49.8%) as shown in Fig. 2b. The stage II 3-year OS rate was not different between the two groups (ES, 81.6%; BTS, 75.0%) as shown in Fig. 2c, whereas stage III 3-year OS rate was different between the two groups (ES, 70.7%; BTS, 49.8%) as shown in Fig. 2d. These results suggest that vascular invasiveness and pathological node-positive status were correlated with tumor progression after SEMS placement; thus, the survival rate was affected by poor prognosis in the BTS group.

**Table 4:** Comparison of pathological characteristics of emergency surgery and bridge to surgery

		ES (*n* = 67)	BTS (*n* = 50)	*p*
pT factor	T4b/ T4a/ T3	12/ 16/ 39	3/ 11/ 36	0.131
Total Lymph nodes	Median (range)	16 (3–52)	21 (4–58)	0.062
pN factor	N0/ N1/ N2/ N3	37/ 20/ 7/ 3	19/ 17/ 13/ 1	0.134
Histological	tub1/ tub2/ others	12/ 51/ 4	15/ 35/ 0	0.126
Lymphatic invasion	ly 0/1/2/3	21/ 33/ 14/ 0	0/ 29/ 16/ 5	< 0.001
Venous invasion	v 0/1/2/3	14/ 31/ 22/ 1	4/ 23/ 17/ 6	0.038
Surgical clearance	Cur A/ B/ C	67/ 0/ 0	50/ 0/ 0	–

Results of adjusted multiple Cox proportional hazard regression for RFS and OS in all stages and stage III disease are presented in Table 5. After adjusting for possible confounders, venous invasion and BTS independently predicted poor RFS in all stages, and venous invasion independently predicted poor RFS in stage III disease. Venous invasion and BTS were also significantly associated with OS in stage III disease.

**Table 5:** Multivariate analysis of recurrence-free survival at all Stages and Stage III

Variables	All Stages Hazard ratio ± SD (95% CI)	*p*-value	Stage III Hazard ratio ± SD (95% CI)	*p*-value
Recurrence free suvival				
ES vs SEMS	HR 0.42 ± 0.20 (0.03, 0.82)	**0.036**	HR 0.45 **±** 0.26 (−0.04, 0.98)	0.071
pT factor; T3/T4	HR −0.55 ± 0.25 (−1.03, −0.06)	0.089	HR −0.51 ± 0.31 (−1.13, 0.13)	0.258
pN factor; N0/N1/N2–3	HR − 0.39 ± 0.29 (− 0.97, 0.14)	0.364	HR − 0.03 ± 0.23 (− 0.49, − 0.43)	0.891
Verous invasion (v0–1/v2–3)	HR − 0.57 ± 0.19 (− 0.96, − 0.21)	**0.002**	HR −0.59 ± 0.24 (− 1.11, − 0.14)	**0.001**
Lymphatic invasion (ly0–1/ly2–3)	HR 0.19 ± 0.22 (− 0.24, 0.64)	0.396	HR 0.01 ± 0.24 (− 0.40, 0.56)	0.752
Overall suvival				
ES vs SEMS	HR 0.86 ± 0.22 (0.42, 1.32)	**< 0.001**	HR 0.99 ± 0.31 (0.40, 1.64)	**< 0.001**
pT factor; T3/T4	HR −0.86 ± 0.28 (− 1.41, 0.31)	**0.007**	HR − 1.21 **±** 0.40 (−2.03, − 0.42)	**0.019**
pN factor; N0/N1/N2–3	HR − 0.40 ± 0.30 (− 1.01, 0.18)	0.358	HR − 0.01 ± 0.24 (− 0.49, − 0.49)	0.976
Verous invasion (v0–1/v2–3)	HR − 0.37 ± 0.20 (− 0.77, − 0.02)	0.065	HR −0.57 ± 0.27 (− 1.13, − 0.07)	**0.025**
Lymphatic invasion (ly0–1/ly2–3)	HR 0.21 ± 0.23 (− 0.23, 0.69)	0.348	HR 0.29 ± 0.26 (− 0.21, 0.81)	0.253

## Discussion

Acute colonic obstruction requires emergent surgical intervention, a mandatory conventional treatment skill. Emergent surgical treatment is associated with high morbidity, mortality, and stoma creation rates, affecting the quality of life of patients. Malignant colorectal obstruction is not only an intestinal obstruction but also an advanced stage CRC. Their prognosis was poorer than that in patients with non-occlusive disease because of highly invasiveness and distant metastasis [25, 26]. Chen et al. revealed that the prognosis in patients with perforation associated with obstruction was poor [13]. Early intervention in the clinical setting before the colonic perforation has been established. Endoscopic placement of colonic stents improves the high decompression effect and reduces clinical symptoms [10].

High postoperative complication rates were correlated with poor prognosis in patients with cancer in several organs [27–30]. Reducing complication rates can improve the prognosis. Our results showed high clinical success rate after SEMS placement and high primary anastomosis rate. Stent-related complications required emergent surgical interventions; however, the stent placement is safe and feasible in this study. Moreover, the laparoscopic rate was high, and postoperative complication rate was 10%. Clinical results, including short-term outcomes in BTS after SEMS, were verified through a meta-analysis [9, 10, 15].

The prognosis was poor in patients with stent perforation and increased local recurrence rate after the colonic stent placement [20]. However, the long-term prognosis in patients with colorectal obstruction after BTS was not different compared with that in patients without obstruction [31–34]. According to the European Society of Gastrointestinal Endoscopy clinical guideline that considers the risk of perforation due to colorectal stents, only limited uses are allowed; therefore, colorectal stent placement is not a standard treatment [35–38]. The prognostic outcomes of BTS in this study were significantly worse than those of ES. Particularly, in lymph node-positive patients, lymphatic and venous invasion seemed to be a significant prognostic factor**.** Although reduced postoperative complication rate would improve the prognosis, our results were contradictory after the stent replacement. These results suggested that stent placement leads to poor prognosis. A concern that colonic stents may be associated with adverse effects of mechanical expansion also exists [39, 40]. Mechanical expansion may be associated with the growth of solid tumors, particularly lymphatic and venous invasion [41, 42].

We found that recurrence and OS were associated with high vascular invasion after a colonic stent placement. Venous invasion was an independent factor for recurrence and prognosis. The CK20 mRNA level, an epithelial marker, is significantly increased in peripheral blood serum, suggesting stent deployment into the vasculature [43]. Alliteratively, Ki-67 level, associated with cellular proliferation, and p27 gene, assisting cell cycle progression, were measured using specimens obtained before and after SEMS insertion; next, the Ki-67 level decreased in the specimen after an SEMS placement compared with that before, and cell proliferation was suppressed [44]. The prognostic nutritional index and serum albumin levels were significantly decreased after stenting, suggesting its disadvantage as BTS [45]. The duration from stent placement to surgery was 14 days. Oncological and nutritional factors might change in the blood and contribute to poor prognosis during the waiting period. Mechanical expansion of the replacement should be minimized to prevent perforation and molecular cytological factors. To improve the materials, expansion and establishment of new mechanism are necessary in colorectal obstruction [46, 47].

These findings should be considered in light of several limitations. First, this is a retrospective, non-randomized, small sample sized study from a single institution; thereby, the heterogeneity of the surgical strategy may have affected the prognostic factors. Second, although validated endoscopic procedures were validated, stent devices used in this study had different lengths, types, and thickness and obtained from different vendors. Lastly, we performed stent placement in the patients with CROSS 3 and 4 who are not indicated for stent insertion until 2015.

To investigate the oncological long-term prognosis of colonic SEMS placement as a bridge to elective surgery, large sample size and prospective randomized controlled studies are warranted to develop a treatment strategy for CRC with obstruction.

## Conclusion

Vascular invasiveness was correlated with tumor progression after a SEMS placement, and OS and RFS rates were lower in BTS. SEMS placement potentially worsens prognostic outcomes in stage II–III malignant colorectal obstruction.

## Data Availability

The datasets used and analyzed during this study are available from the corresponding author upon reasonable request.

## Abbreviations

BTS: Bridge to surgery
CRC: Colorectal cancer
CROSS: ColoRectal Obstruction Scoring System
ES: Emergency surgery
ESGE: European Society of Gastrointestinal Endoscopy
JSCCR: Japanese Society for Cancer of the Colon and Rectum
RECIST 1.1: Response Evaluation Criteria in Solid Tumors, version 1.1
OS: Overall survival
RFS: Recurrence-free survival
SEMS: Self-expandable metallic stent
WSES: World society of emergency surgery
